# Highly Reversible Li–Se Batteries with Ultra-Lightweight N,S-Codoped Graphene Blocking Layer

**DOI:** 10.1007/s40820-018-0213-5

**Published:** 2018-06-28

**Authors:** Xingxing Gu, Lingbao Xin, Yang Li, Fan Dong, Min Fu, Yanglong Hou

**Affiliations:** 1 Chongqing Key Laboratory of Catalysis and New Environmental Materials, College of Environment and Resources, Chongqing Technology and Business University, Chongqing, 400067 People’s Republic of China; 2 School of Chemical Engineering, Shandong University of Technology, Zibo, 255049 Shandong People’s Republic of China; 3 College of Arts, Chongqing Technology and Business University, Chongqing, 400067 People’s Republic of China; 4 Beijing Key Laboratory for Magnetoelectric Materials and Devices (BKLMMD), BIC-ESAT, Department of Materials Science and Engineering, College of Engineering, Peking University, Beijing, 100871 People’s Republic of China

**Keywords:** Li–Se batteries, N,S-codoped, Graphene, Ultra-lightweight, Free-standing, Vacuum filtration

## Abstract

The desire for practical utilization of rechargeable lithium batteries with high energy density has motivated attempts to develop new electrode materials and battery systems. Here, without additional binders we present a simple vacuum filtration method to synthesize nitrogen and sulfur codoped graphene (N,S-G) blocking layer, which is ultra-lightweight, conductive, and free standing. When the N,S-G membrane was inserted between the catholyte and separator, the lithium–selenium (Li–Se) batteries exhibited a high reversible discharge capacity of 330.7 mAh g^−1^ at 1 C (1 C = 675 mA g^−1^) after 500 cycles and high rate performance (over 310 mAh g^−1^ at 4 C) even at an active material loading as high as ~ 5 mg cm^−2^. This excellent performance can be ascribed to homogenous dispersion of the liquid active material in the electrode, good Li^+^-ion conductivity, fast electronic transport in the conductive graphene framework, and strong chemical confinement of polyselenides by nitrogen and sulfur atoms. More importantly, it is a promising strategy for enhancing the energy density of Li–Se batteries by using the catholyte with a lightweight heteroatom doping carbon matrix.

**Electronic supplementary material:**

The online version of this article (10.1007/s40820-018-0213-5) contains supplementary material, which is available to authorized users.

## Highlights

- A free-standing, ultra-lightweight, N,S-codoped graphene membrane is assembled by a simple vacuum filtration method.
- The N,S-codoped graphene membrane is first used as the blocking layer for a polyselenide catholyte.
- The Li–Se batteries based on the as-prepared graphene membrane exhibites excellent cycling performance and rate capability at high selenium loading (5 mg cm^−2^).

## Introduction

Rechargeable lithium–selenium (Li–Se) batteries have recently attracted considerable attention as potential energy storage devices for portable electronics and electric vehicles because Se has a high volumetric capacity (3253 mAh cm^−3^), which is comparable to that of sulfur (3467 mAh cm^−3^) [1–5], and has a relatively high electronic conductivity among nonmetallic materials (1 × 10^−3^ S m^−1^) [2, 6]. Despite these advantages of Li–Se batteries, great challenges regarding the cathode impede their practical applications. Similar as the Li–S batteries, these challenges of Li–Se batteries mainly involve the dissolution of polyselenide intermediates [2, 7, 8] and electrode collapse [2], which result in considerable loss of the active material and rapid capacity decay [9–12].

To overcome these issues, a common approach, trapping selenium/sulfur in porous carbon hosts [1, 2, 8, 12–18], has been widely used. The resulting conductive and porous structure could provide electronic conductivity while hosting selenium and its discharge products [19]. Owing to the physical confinement of selenium in porous carbon, the lithium polyselenide shuttle phenomenon could be greatly suppressed; in addition, the cathode could retain its integrity very well, leading to improved cycling stability and Coulombic efficiency. However, owing to the nonpolar nature of carbon, the interaction between carbon and lithium polyselenides is relatively weak, causing a gradual loss of the polar polyselenides during cycling. This phenomenon is even worse for cells with high active material loading in the cathodes. Thus, a more effective approach using chemical adsorption of lithium polyselenides has been reported recently [20–24]. For instance, our previous work using a nitrogen-doped loofah sponge carbon interlayer in Li–Se batteries illustrated that polyselenides could be effectively retained by N atoms [24]. Wen’s group designed a conductive heterocyclic selenized polyacrylonitrile compound by the dehydrogenation/selenation method at high temperature [23, 25]. This conductive selenized polymer material could perform stably over thousands of cycles with a higher specific capacity (> 300 mAh g^−1^) and exhibited a considerably better rate capability compared to conventional oxides [23]. Zhang et al. [20] used first-principle calculations to evaluate the influence of heteroatom doping on the electrochemical performance and confirmed that the presence of heteroatoms in the carbon framework greatly facilitates the interaction between carbon and Li_2_Se.

Though significantly improved cycling stability has been obtained by using these porous (heteroatom) carbon and carbon interlayers, it should be noted that the excellent cycling performance is achieved at relatively low selenium contents (less than 60%) and low selenium areal loading (< 2 mg cm^−2^) in the final cathode [1, 4, 8, 13, 26–28]. There is no doubt that the overall energy density of Li–Se batteries would be seriously reduced if these cathodes with low selenium content and low selenium areal loading are used. Therefore, advanced architectures and abundant functional groups/heteroatoms are needed to ensure simultaneous realization of high selenium content and high selenium areal loading with excellent electrochemical performance.

**Fig. 1:**
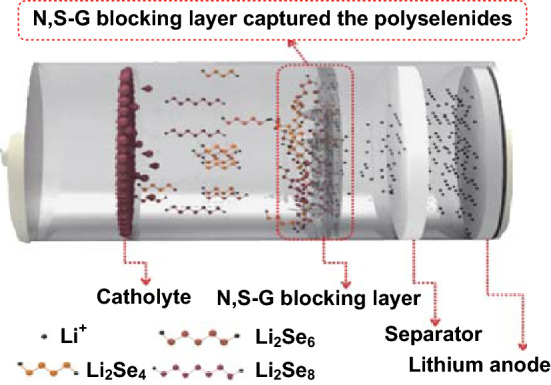
N,S-G membrane as an interlayer for trapping polyselenides

In this work, we present a new strategy to improve the cycling stability of Li–Se batteries with high active material loading by using ultra-lightweight, free-standing, N,S-codoped graphene (N,S-G) membranes as both current collectors and blocking layers. As an interlayer for Li–Se batteries (a schematic is shown in Fig. 1), the conductive N,S-G membrane (conductivity 148 S m^−1^) could, on the one hand, enhance the electron and lithium ion conductivity to achieve high selenium utilization even in cells with a very high selenium areal loading of 5 mg cm^−2^; on the other hand, it could effectively restrict polyselenide shuttling via physical/chemical inhibition to afford a stable cycle life.

## Experimental

### Material Synthesis

#### Preparation of N,S-G

The N,S-G was prepared according to our previous work [29]. The detailed synthesis processes are as follows: First, 20 mg of thiocarbohydrazide (analytical reagent, Sigma-Aldrich) were added to 5 mL of a graphene oxide (GO) suspension with a concentration of 5 mg mL^−1^. Subsequently, the mixture was held in an oil bath at 90 °C without stirring for 30 min after 1 min of sonication. After the solution cooled naturally to room temperature, the resulting N,S-G hydrogels with cylindrical structure were immersed in deionized water for 24 h and washed several times to remove residual impurities. Finally, the hydrogels were vacuum-dried overnight for further processing.

#### Free-Standing N,S-G Membrane Preparation

N,S-G (7.5 mg) was dispersed in 5 mL of an *N*-methylpyrrolidone solution under sonication for 1 h. Subsequently, the homogenous N,S-G solution was vacuum-filtrated with a Celgard 2300 separator as the filter membrane. Then, the entire filter membrane was placed in a vacuum oven and dried overnight at room temperature. The N,S-G could then be peeled from the filter. After being peeled off, the N,S-G membrane was continuously vacuum-dried for another 12 h at 60 °C. The diameter of the N,S-G membrane was approximately 3.8 cm.

#### Preparation of Li_2_Se_8_ Solution

Li_2_Se (0.186 g, Shanghai Longjin Metallic Material Co., Ltd.) and 1.106 g of selenium (analytical reagent, Sigma-Aldrich) were added to 10 mL of 1 M lithium bis(trifluoromethanesulfonyl)imide solution, which uses 1,3 dioxolane/1,2-dimethoxyethane (1:1 v/v) as the solvent and contains 0.1 M LiNO_3_. Then, the mixed solution was stirred at 80 °C for 24 h to obtain the 0.2 mol L^−1^ Li_2_Se_8_ electrode solution. All the operations were conducted in a glove box under Ar atmosphere.

## Material Characterization

The samples’ structures were characterized by X-ray diffraction (XRD, Model LabX-6000, Shimadzu, Japan), X-ray photoelectron spectroscopy (XPS, Kratos Analytical Ltd., Manchester, UK), and Raman scattering (Renishaw Inc., Illinois, USA). The morphologies of the obtained samples were characterized by transmission electron microscopy (TEM, Tecnai 20 FEI, USA) with an acceleration voltage of 200 kV. The membrane morphologies were investigated by scanning electron microscopy (SEM, JSM-7001F, JEOL, Japan).

## Electrochemical Measurements

The large free-standing N,S-G membrane was cut into small wafers (1.4 cm in size and approximately 1.0 mg in weight). The small wafers were used as the current collector and blocking layer. A 0.2 M Li_2_Se_8_ solution was used directly as the catholyte. To a 2032 coin cell, 40 μL of Li_2_Se_8_ solution was added to the surface of the N,S-G wafers or directly to the surface of the Celgard 2300 separator to obtain a selenium loading of ~ 5 mg cm^−2^ when the weight of N,S-G membrane in the electrode is considered. The charge/discharge performance of the coin cells was tested using a LAND CT-2001A instrument (Wuhan, China), and the potential range was controlled between 1.5 and 3.0 V at room temperature. A CHI 660D electrochemical workstation (CHI Instrument, Shanghai, China) was used to perform cyclic voltammetry (CV) measurements at a scan rate of 0.1 mV s^−1^ and a potential of 1.5–3.0 V. Electrochemical impedance spectroscopy (EIS) was also performed using the same instrument over a frequency range of 100 kHz–10 MHz. The electrical conductivity of the N,S-G membrane was determined by a four-point probe method on a resistivity measurement system (RTS-8, China).

## Results and Discussion

**Fig. 2:**
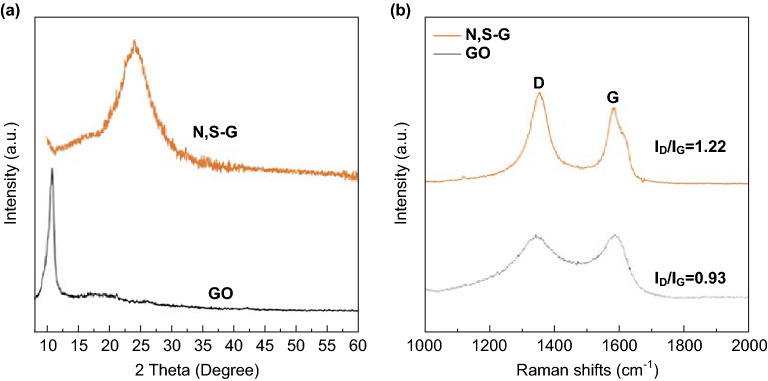
**a** XRD patterns of GO and N,S-G. **b** Raman spectra of GO and N,S-G

In the preparation process, thiocarbohydrazide not only acted as the reductant to reduce the GO to graphene; it also introduced nitrogen (C–N, –NH_2_) and sulfur (C–S, –SO_3_H) atoms into the graphene structure to produce N,S-G [29]. As shown in Fig. 2a, the in situ redox reaction of GO to N,S-G can be confirmed by XRD analysis. The pristine GO shows its characteristic X-ray powder diffraction peak at 10.5°. However, for the N,S-G sample, the peak at 10.5° disappears, and instead a new, broader peak at around 25.3° is observed, suggesting successful reduction from GO to graphene [30]. Further, the Raman spectrum, as shown in Fig. 2b, also confirms the reduction of GO to graphene. The D band corresponds to the structural defects and disorder in the carbon matrix. The G band is related to the *E*_2g_ vibrational mode of *sp*^2^-bonded carbon atoms [30]. As we can observe, the *I*_D_/*I*_G_ ratio of N,S-G is notably larger than that of GO (1.22 vs. 0.93); the reason is that the heteroatom doping and reduction increased the defects in the obtained N,S-G [30, 31]. Furthermore, the G band of the N,S-G is downshifted to 1584 cm^−1^ and the D band is upshifted to 1354 cm^−1^ compared with those of GO (~ 1594 and ~ 1350 cm^−1^, respectively), which is attributed to doping with nitrogen and sulfur atoms [32].

**Fig. 3:**
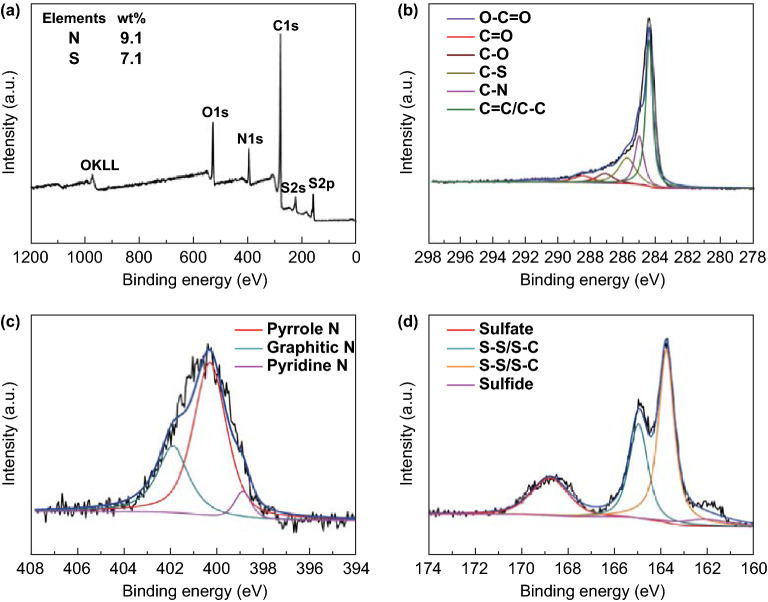
**a** XPS spectrum of N,S-G and high-resolution **b** C 1*s*, **c** N 1*s*, and **d** S 2*p* spectra

The nitrogen and sulfur atom doping can be further detected by XPS characterization. As shown in Fig. 3a, the XPS spectrum of N,S-G reveals that the N and O contents are 7.1 and 9.1 at.%, respectively. Figure 3b shows the high-resolution XPS C 1*s* core-level spectrum. The peak centered at 284.5 eV can be attributed to C–C/C=C in *sp*^2^-hybridized domains, whereas the peaks at 286.1, 286.8, and 288.5 eV are related to C–O epoxy and hydroxyl groups, C=O carbonyl groups, and O=C–O carboxyl groups, respectively [33]. The new peaks at 286.0 and 285.1 eV reveal the formation of C–S and C–N bonds [29]. The doped nitrogen atoms in the C–N bonds exist in three different states: pyridinic nitrogen (398.1 eV), pyrrolic nitrogen (399.6), and graphitic nitrogen (401.6), as shown in Fig. 3c, indicating that nitrogen is successfully doped into the graphene framework rather than present as a residue or impurities [29, 30]. Figure 3d shows the high-resolution XPS S 2*p* core-level spectrum. Four peaks can be observed, which are attributed to S–S and S–C bonds (163.5 and 164.9 eV, respectively), sulfide (162.1 eV), and sulfate (168.8 eV), suggesting successful sulfur doping in the graphene framework [29, 34].

**Fig. 4:**
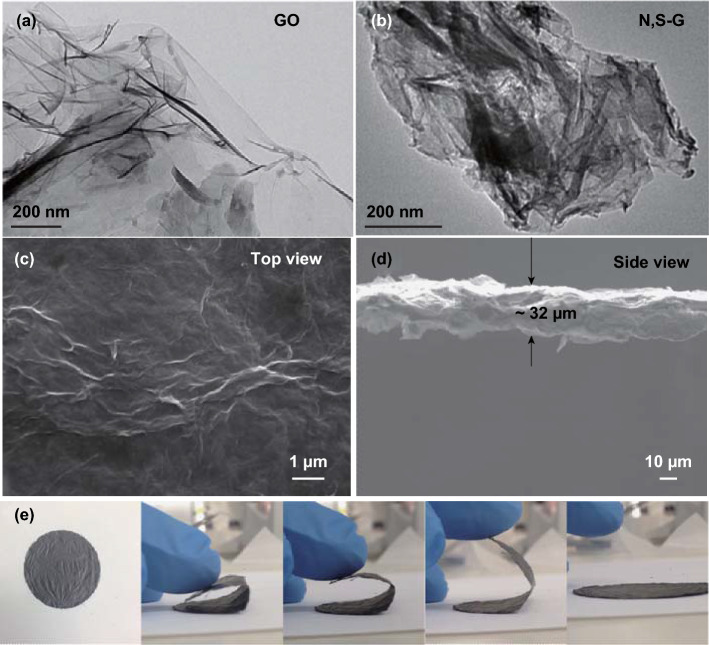
TEM images of **a** GO and **b** N,S-G. SEM images of N,S-G membrane in: **c** top view, **d** side view, and **e** the free-standing N,S-G membrane

The morphology of the as-prepared N,S-G was investigated by TEM. As shown in Fig. 4a, the pristine GO exhibits excellent sheet structure. After reacting with the thiocarbohydrazide at 90 °C, the obtained N,S-G still shows the sheet structure (Fig. 4b) but tends to aggregate slightly. The morphology of the free-standing N,S-G was further revealed by SEM. From the top view (Fig. 4c), it can be observed that numerous sheets interweave to form the membrane structure; from the side view (Fig. 4d), it can be observed that this membrane was self-assembled layer by layer, and its thickness is only approximately 32 μm. Further, due to this assembly pattern, the obtained N,S-G membranes are free-standing and demonstrate good flexibility, as shown in Fig. 4e.

**Fig. 5:**
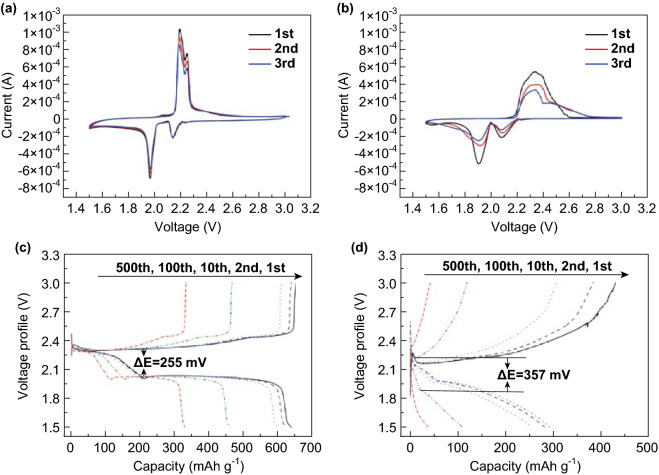
CV curves of the first three cycles for Li–Se batteries **a** with and **b** without N,S-G interlayer at a scan rate of 0.1 mV s^−1^. Galvanostatic charge/discharge voltage profiles of Li–Se batteries **c** with and **d** without N,S-G interlayer at 1 C between 1.5 and 3.0 V

The electrochemical performance of Li–Se batteries with and without the N,S-G interlayer was investigated using CR2032 coin cells. Figure 5a, b shows typical CV curves of the Li–Se batteries with and without the N,S-G interlayer, respectively. The first cathodic scan in Fig. 5a reveals two reduction peaks at 2.15 and 1.97 V, which correspond to stepwise electrochemical reduction of selenium to polyselenides and finally Li_2_Se [8, 24]. The subsequent anodic scan shows a strong oxidation peak at 2.25 V with a shoulder, indicating reversible conversion of Li_2_Se to polyselenides and even selenium. Even though the CV curves in Fig. 5b also show two reduction peaks and one split oxidation peak, the peaks become very broad, and they do not overlap well, all of which suggests intensive polarization and poor stability of the polyselenide catholyte without the N,S-G interlayer [8, 24]. In other words, the N,S-G interlayer is beneficial for stabilization of the polyselenide catholyte.

Figure 5c, d shows the galvanostatic charge/discharge voltage profile of the Li–Se batteries with and without the N,S-G interlayer at different cycles at a current density of 1 C between 1.5 and 3.0 V. There are two voltage plateaus in the discharge process for the catholyte with the N,S-G interlayer (Fig. 5c): a short plateau at 2.25 V and a long plateau at 1.97 V, which could be attributed to a series of reduction reactions from high-order polyselenides to low-chain Li_2_Se. In addition, a long charge plateau at approximately 2.25 V and a short one at approximately 2.4 V are observed in the charge process; these plateaus correspond to the reverse reaction from Li_2_Se to high-order polyselenides and even to elemental selenium. Both the charge and discharge voltage plateaus are in good agreement with the CV measurements. Further, even after 500 cycles, the two voltage plateaus are still clearly observed. However, the voltage plateaus are barely observed for the catholyte without the N,S-G interlayer, as shown in Fig. 5d. In addition, the cell with the N,S-G interlayer exhibits a low polarization of 255 mV (vs. 357 mV for the cell without the N,S-G interlayer) after 100 cycles at 1 C. Further, the catholyte without the N,S-G interlayer also reveals strong overcharge in different cycles. These distinct charge/discharge characteristics further confirm the lower polarization, higher electrochemical stability, and reversibility of the polyselenide catholyte with the N,S-G interlayer [8].

**Fig. 6:**
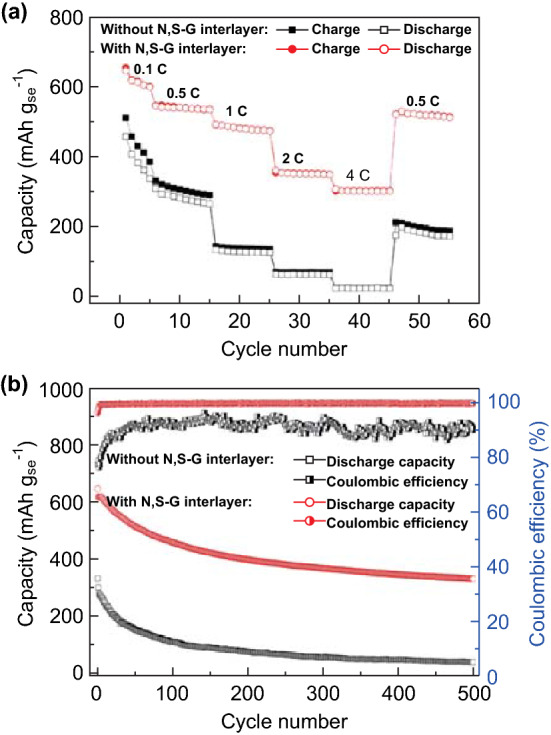
**a** Rate performance of Li–Se cells with and without N,S-G interlayer. **b** Long-term cycling performance of Li–Se cells with and without N,S-G interlayer

The cycling performance of the cells with and without the N,S-G interlayer at various C rates between 1.5 and 3.0 V is compared in Fig. 6a. The initial discharge capacity of the cell with the N,S-G interlayer is as high as 648.2 mAh g^−1^; this corresponds to a selenium utilization of 95.6%, which is much higher than that of the cell without the interlayer (48.9%), further confirming the excellent redox reaction kinetics and reversibility of the Li–Se batteries with the N,S-G interlayer system [19]. Moreover, reversible capacities of 534.6, 473.5, 348.6, and 301.4 mAh g^−1^ could be achieved at C rates of 0.5 C, 1 C, 2 C, and 4 C, respectively, for the cell with the interlayer. In addition, when the current density was switched to 0.5 C, a reversible discharge capacity of 512.2 mAh g^−1^ could be recovered, again indicating the good stability of the selenium cathode with the interlayer. It is clearly observed that the rate capability of the catholyte with the N,S-G interlayer is far better than that of the catholyte without the N,S-G interlayer. Moreover, we compared the obtained performance with those reported in previous works, as shown in Table S1; the table reveals that the Li–Se batteries with the N,S-G interlayer showed excellent cycle life span and rate performance at a high selenium loading.

To further demonstrate the advantages of inserting the N,S-G interlayer, the long-term cycling performance of the cells with and without the interlayer was evaluated at a rate of 1 C for 500 cycles. As shown in Fig. 6b, the cell with N,S-G interlayer has a high initial discharge capacity of 638.5 mAh g^−1^ and a reversible capacity of 330.7 mAh g^−1^ after 500 cycles. The capacity decay is as low as 0.09% per cycle, which is half that without interlayer. Further, to the best of our knowledge, this is one of the best cycling performances among cathodes with high selenium areal loading, as shown by the comparison in Table S1. In addition, the corresponding Coulombic efficiency of the cell with the N,S-G interlayer is still as high as 99.6% after 500 cycles, whereas that of the catholyte in the cell without the N,S-G interlayer is only 90.5%, demonstrating that the active materials are well confined within the N,S-G interlayer by the effective chemisorption and physical adsorption of lithium polyselenides [19, 24, 35].

**Fig. 7:**
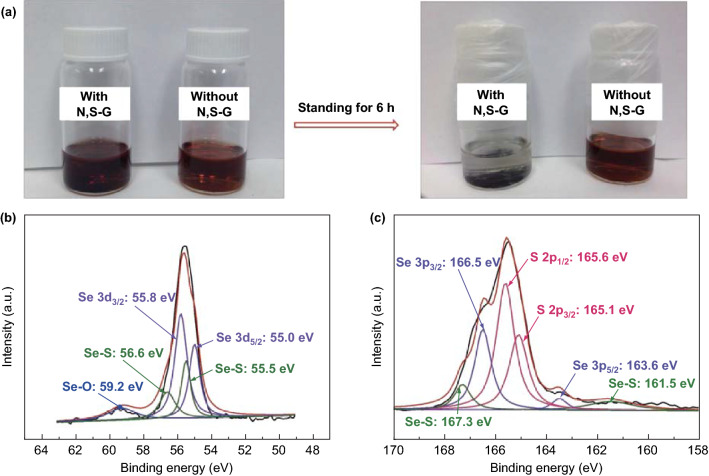
**a** Digital photographs of polyselenide adsorption by N,S-G interlayers. High-resolution **b** Se 3*d* and **c** S 2*p* XPS spectra of N,S-G interlayer after 500 discharge cycles

In order to better understand the positive role of the N,S-G interlayer in the Li–Se batteries, we first investigated the morphology of N,S-G after discharging. As seen in the SEM image in Fig. S1, very few large particles can be found on the surface of the discharged N,S-G interlayer, and the elements of Se, N, and S are distributed homogenously in the corresponding element map; these results both indicate that the discharge products are uniformly precipitated on the surface of N,S-G. The uniform precipitation of the discharge products could reduce the contact resistance between the active materials and carbon matrix and thus increase the Li^+^-ion accessibility, electronic transport, and selenium utilization [19]. This homogenous dispersion could be attributed to the strong interactions between the polar lithium polyselenides and polar N, S, and O functional groups in the N,S-G framework [19]. We also conducted simple adsorption experiments in a glove box under Ar atmosphere, as shown in Fig. 7a. It can be clearly observed that, after soaking for 6 h, the Li_2_Se_8_ solution becomes nearly transparent with the addition of N,S-G, whereas the solution without N,S-G retained its original brownish red color, which indicates that the Li_2_Se_8_ was adsorbed by N,S-G. To further confirm the chemical interactions of the lithium polyselenides with N, S, and even O functional groups, the high-resolution Se 3*d* and S 2*p* XPS spectra of the N,S-G interlayer (Fig. 7b, c) after 500 discharge cycles were analyzed. It can be observed that the Se–O bond appears at 59.3 eV in the Se 3*d* spectrum in Fig. 7b [6, 9, 36], and the Se–S bond appears at ∼ 55.5/∼ 56.6 eV in the Se 3*d* spectrum in Fig. 7b and at 161.5/167.3 eV in the S 2*p* spectrum in Fig. 7c [35, 37]. Additionally, in both our previous work [24] and work by Shi’s group [35], first-principle calculations based on density functional theory were performed to verify that the N atoms provide strong chemical adsorption of the lithium polyselenides.

Finally, the EIS spectra were also obtained to demonstrate that the N,S-G interlayer is beneficial for improving the electrochemical performance. The impedance spectra (Fig. S2) were analyzed by fitting to an equivalent circuit (inset of Fig. S2). Before cycling, there is only one semicircle following a slope line in the impedance spectrum. The corresponding equivalent circuit consists of an electrolyte resistance (*R*_e_); a constant phase element (CPE) in parallel with an ohmic resistance (*R*_ct_), which represents the impedance of lithium ion transport through the surface film and charge transfer at the electrode/electrolyte interface; and a Warburg element (*W*_0_) in series, which accounts for the lithium ion diffusion inside the active materials [9]. Obviously, the polyselenide catholyte with the N,S-G interlayer shows a much lower combined resistance *R*_ct_ than the catholyte without the N,S-G interlayer (38.64 vs. 176.80 Ω, Table S2), demonstrating better electrical contact with the electrode after the conductive interlayer is inserted. After 500 charge/discharge cycles, one more semicircle appears at high frequency in the impedance spectrum. Further, the corresponding equivalent circuit exhibits an extra constant phase element (CPE1) in parallel with an ohmic resistance (*R*_s_) associated with the solid electrolyte interface film [26]. The *R*_ct_ value of the electrode with the N,S interlayer is still clearly much lower than that of the electrode without the interlayer (65.92 vs. 479.10 Ω, Table S2). In order to further confirm that the conductive N,S-G membrane could significantly enhance the redox reaction kinetics, the Li^+^ ion diffusion coefficients of the cells with and without the N,S-G interlayer were compared. *Z*_re_ is calculated by Eq. 1 [38, 39]:

1 $$\left| {Z_{\text{re}} } \right| = R_{\text{s}} + R_{\text{ct}} + \sigma_{\text{w}} \omega^{ - 0.5}$$

**Fig. 8:**
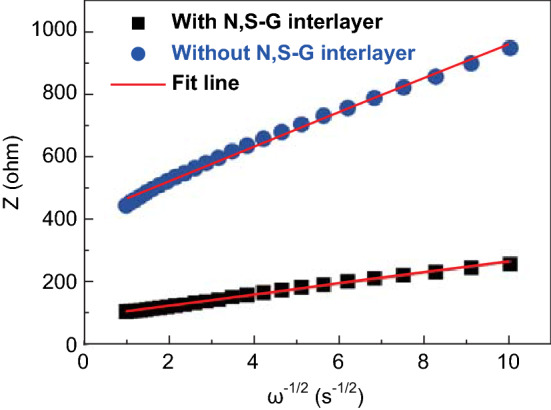
*Z* as a function of *ω*^−1/2^ in the low-frequency region

The Warburg coefficient (*σ*_w_) is calculated by plotting *Z*_re_ versus the reciprocal square root of the low angular frequencies (*ω*) according to the EIS spectrum in Fig. S2b. The slope of the fitted line is the value of *σ*_w_, as shown in Fig. 8. Because the Li^+^ diffusion coefficient (*D*_Li_) is inversely proportional to *σ*_w_, *D*_Li_ is further calculated by Eq. 2 [38, 40]:

2 $$D_{\text{Li}} = \frac{{R^{2} T^{2} }}{{2A^{2} n^{4} F^{4} c^{2} \sigma_{\text{w}}^{2} }}$$

where *R* is the gas constant, *T* is the absolute temperature, *A* is the electrode surface area, *n* is the number of electrons per molecule during the electrochemical reaction, *F* is the Faraday constant, and *c* is the Li^+^ concentration.

After 500 charge/discharge cycles, the calculated values of *σ*_w_ of Li–Se cells with and without the N,S-G interlayer are 17.7 and 55.0, respectively, and the corresponding *D*_Li_ value of the Li–Se cell with the interlayer is 9.6 times higher than that of the cell without the N,S-G interlayer, indicating that the N,S-G blocking layer is favorable for the redox reaction kinetics [38–40].

Moreover, the polyselenide catholyte with the N,S-G interlayer also shows a smaller *R*_s_ value (34.23 vs. 195.70, Table S2), which indicates that the N,S-G interlayer could effectively prohibit lithium polyselenide shuttling to the anode to form a Li_2_Se/Li_2_Se_2_ film [24].

## Conclusions

A free-standing and ultra-lightweight N,S-G membrane was successfully prepared through a facile and simple vacuum filtration method. When it was used as an interlayer for a polyselenide catholyte, the corresponding Li–Se cells exhibited a high specific capacity and excellent rate and cycling performance even at a high selenium content (79 wt%) and high selenium loading (5 mg cm^−2^). A reversible discharge capacity of 330.7 mAh g^−1^ was obtained at 1 C after 500 cycles. This superior electrochemical performance compared to the cell without the N,S-G interlayer could be attributed to good dispersion of the liquid active material in the electrode, high Li^+^-ion accessibility, fast electronic transport in the conductive graphene framework, and strong chemical adsorption of polyselenides. This work may provide a new route toward optimization of the carbon matrix for high-energy-density Li–Se batteries.

## Electronic supplementary material

Below is the link to the electronic supplementary material.

Supplementary material 1 (PDF 478 kb)
