# Supplementary material for: Highly Reversible Li–Se Batteries with Ultra-Lightweight N,S-Codoped Graphene Blocking Layer
Source: Nanomicro Lett. 2018 Jun 28;10(4):59. doi: 10.1007/s40820-018-0213-5 (PMC6199105; doi:10.1007/s40820-018-0213-5)
Supplement: Supplementary file 1 — Supplementary material 1 (PDF 478 kb) [file 40820_2018_213_MOESM1_ESM.pdf]

Supporting Information for

Highly Reversible Li–Se Batteries with Ultra-Lightweight N,S-Codoped Graphene Blocking Layer

Xingxing Gu[1,*], Lingbao Xin[2], Yang Li[3], Fan Dong[1], Min Fu[1], Yanglong Hou[4,*]

[1]Chongqing Key Laboratory of Catalysis and New Environmental Materials, College of Environment and Resources, Chongqing Technology and Business University, Chongqing 400067, People's Republic of China

[2]School of Chemical Engineering, Shandong University of Technology, Zibo, Shandong 255049, People's Republic of China

[3]College of Arts, Chongqing Technology and Business University, Chongqing 400067, People's Republic of China

[4]Beijing Key Laboratory for Magnetoelectric Materials and Devices (BKLMMD), BIC-ESAT, Department of Materials Science and Engineering, College of Engineering, Peking University, Beijing 100871, People's Republic of China

*Corresponding authors. E-mail: x.gu@ctbu.edu.cn (Xingxing Gu), hou@pku.edu.cn (Yanglong Hou)

Supplementary Figures

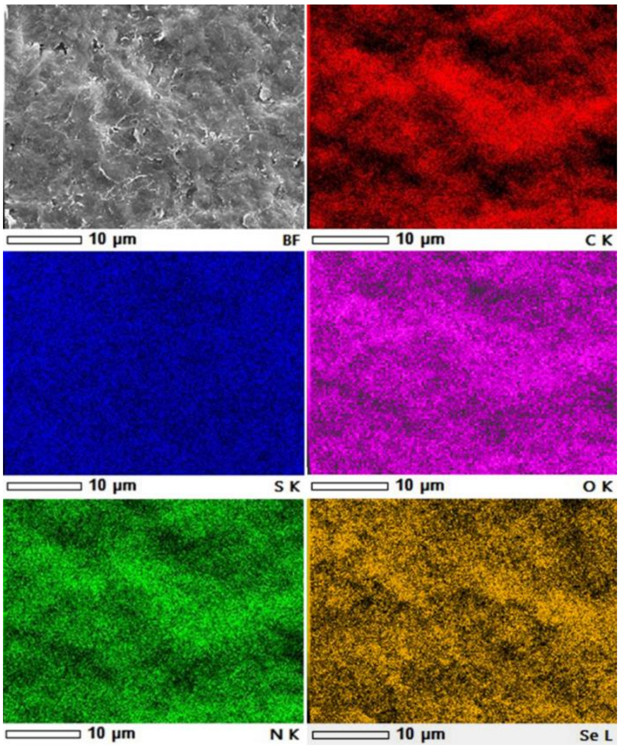

Fig. S1 SEM images of the N,-S-G interlayer after 500 cycles and the corresponding element mapping of C, S, O, N, Se

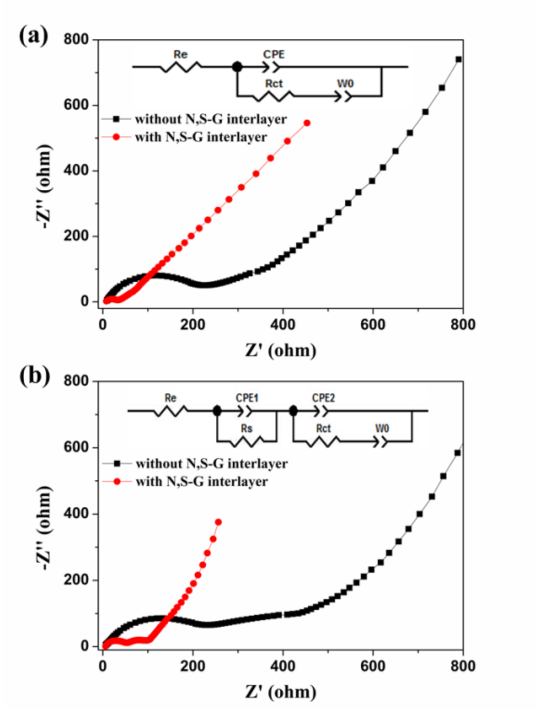

Fig. S2 Nyquist plots and equivalent circuit (inset) of the polyselenides catholyte with and without N,S-G interlayer: **a** before cycling and **b** after 500 cycles

Table S1 Electrochemical performances comparison with different carbon-Se composites cathode and carbon interlayer

Sample name	Selenium content in the cathode (%)	Se loading (mg cm^{-2})	Long-cycle performances	Rate capability	References
polyselenides catholyte with N,S-G interlayer	~79 (including N,S-G weight)	~5.0	330.7 mAh g^{-1} after 500 cycles at 1 C	301.4 mA h g^{-1} at 4 C	This work
Se/porous carbon microcubes composite	40	1.0-1.5	231.4 mAh g^{-1} after 460 cycles at 0.5 C	218.1 mAh g^{-1} at 5 C	[1]
Se-impregnated hollow carbon microspheres	41.7	0.72	525 mAh g^{-1} after 1000 cycles at 0.74 C	496 mAh g^{-1} at 2.97 C	[2]
Se/CNT@microporous carbon composite	50.2	2.0	~200 mAh g^{-1} after 1000 cycles at 1 C	~150 mAh g^{-1} at 5 C	[3]
Graphene-CNT@Se	51	1.6	504.3 mA h g^{-1} after 150 cycles at 0.2 C	436.4 mAh g^{-1} at 4 C	[4]
Se@hollow-core nitrogen-doped carbon nanobelts	62.5	3.0	~453.2 mAh g^{-1} after 1000 cycles at 1 C	~450 mAh g^{-1} at 2.37 C	[5]
Graphene–encapsulated selenium/polyaniline core–shell nanowire	52.9	3.0	461.7 mAh g^{-1} after 200 cycles at 0.2 C	510.9 mAh g^{-1} at 2 C	[6]
Porous carbon nanofibers-selenium	52.3	0.8	516 mAh g^{-1} after 900 cycles at 0.74 C	306 mAh g^{-1} at 5.9 C	[7]

Se/chitosan-derived hierarchical porous carbon	35	1.37-1.77	537.6 mAh g^{-1} after 100 cycles at 0.2 C	325.2 mAh g^{-1} at 4.7 C	[8]
Se/CMK-3	39.2	2.0	600 mAh g^{-1} after 50 cycles at 0.1 C	—	[9]
Se/carbon aerogel	44.8	1.5-2.0	309 mAh g^{-1} after 100 cycles at 0.5 C	301 mAh g^{-1} at 5 C	[10]
Se/porous carbon sphere	56.4	2.0	417 mAh g^{-1} after 1200 cycles at 1 C	384.75 mAh g^{-1} at 20 C	[11]
Se/nitrogen-doped microporous carbon spheres	35	2.0	570 mAh g^{-1} after 350 cycles at 0.5 C	200 mAh g^{-1} at 5 C	[12]
Core-shell structured selenium@ carbon spheres	54.4	1.0-2.0	300 mAh g^{-1} after 100 cycles at 0.1 C	447 mAh g^{-1} at 4.7 C	[13]
Selenium in nitrogen-containing hierarchical porous carbon	45.0	1.0	267 mAh g^{-1} after 100 cycles at 1 C	~300 mAh g^{-1} at 5 C	[14]
Selenium/microporous carbon nanofiber composite	40	1.0−1.2	400 mAh g^{-1} after 2000 cycles at 1 C	420 mAh g^{-1} at 5 C	[15]
Se@Carbide-derived carbon	46.5	1.0	~450 mAh g^{-1} after 150 cycles at 0.2 C	310 mAh g^{-1} at 2 C	[16]
Se@mesoporous carbon/ graphene	49.6	1.2	385 mAh g^{-1} after 1300 cycles at 1 C	274 mAh g^{-1} at 3 C	[17]
Se@erylene-3,4,9,10-tetracarboxylic dianhydride derived carbon	43.2	1.2	430 mAh g^{-1} after 250 cycles at 0.15 C	280 mAh g^{-1} at 1.78 C	[18]
Hollow carbonized polyaniline spheres/selenium composites	32.96	1.5-2.0	298.7 mAh g^{-1} after 100 cycles at 0.5 C	246.8 mAh g^{-1} at 2 C	[19]
Selenium/micro–mesoporous carbon sphere nanocomposite	48	2.0	540 mAh g^{-1} after 100 cycles at 0.1 C	430 mAh g^{-1} at 5 C	[20]
Selenium@mesoporous carbon	38.4	0.55–0.71	306.9 mAh g^{-1} after 100 cycles at 0.5 C	275.9 mAh g^{-1} at 2 C	[21]
Cellulose based carbon interlayer	80 (without carbon interlayer weight)	1.6	282 mAh g^{-1} after 100 cycles at 0.5 C	—	[22]
Se@reduced graphene oxide with CNT interlayer	64% (without CNT interlayer weight)	2.0	265 mAh g^{-1} after 200 cycles at 0.2 C	250 mAh g^{-1} at 5 C	[23]
Graphene-coated polymer separator	52% (including graphene weight)	4.0	331 mAh g^{-1} after 1000 cycles at 0.5 C	~3400 mAh g^{-1} at 5 C	[24]

Table S2 Impedance parameters of the Li-Se batteries calculated according to the equivalent circuits

Sample name	Cycle number	Resistance		
		R_e (Ω)	R_s (Ω)	R_{ct} (Ω)
Without N,S-G interlayer	Before cycling	3.75	—	38.64
	500th cycles	5.87	34.23	65.92
With N,S-G interlayer	Before cycling	7.90	—	176.80
	500th cycles	4.93	195.70	479.10

References

[1] T. Liu, M. Jia, Y. Zhang, J. Han, Y. Li et al., Confined selenium within metal-organic frameworks derived porous carbon microcubes as cathode for rechargeable lithium–selenium batteries. J. Power Sources **341**, 53-59 (2017). https://doi.org/10.1016/j.jpowsour.2016.11.099

[2] Y.J. Hong, Y.C. Kang, Selenium-impregnated hollow carbon microspheres as efficient cathode materials for lithium-selenium batteries. Carbon **111**, 198-206 (2017). https://doi.org/10.1016/j.carbon.2016.09.069

[3] S. Xin, L. Yu, Y. You, H.P. Cong, Y.X. Yin et al., The electrochemistry with lithium versus sodium of selenium confined to slit micropores in carbon. Nano Lett. **16**(7), 4560-4568 (2016). https://doi.org/10.1021/acs.nanolett.6b01819

[4] J. He, Y. Chen, W. Lv, K. Wen, P. Li et al., Three-dimensional hierarchical graphene-CNT@Se: A highly efficient freestanding cathode for Li–Se batteries. ACS Energy Lett. **1**(1), 16-20 (2016). https://doi.org/10.1021/acsenergylett.6b00015

[5] Q. Cai, Y. Li, L. wang, Q. Li, J. Xu et al., Freestanding hollow double-shell Se@CN_x nanobelts as large-capacity and high-rate cathodes for Li-Se batteries. Nano Energy. **32**, 1-9 (2017). https://doi.org/10.1016/j.nanoen.2016.12.010

[6] J. Zhang, Y. Xu, L. Fan, Y. Zhu, J. Liang, Y. Qian, Graphene–encapsulated selenium/polyaniline core–shell nanowires with enhanced electrochemical performance for Li-Se batteries. Nano Energy **13**, 592-600 (2015). https://doi.org/10.1016/j.nanoen.2015.03.028

[7] L.C. Zeng, W.C. Zeng, Y. Jiang, X. Wei, W.H. Li et al., A flexible porous carbon nanofibers-selenium cathode with superior electrochemical performance for both Li-Se and Na-Se batteries. Adv. Energy Mater. **5**(4), 1401377-1401387 (2015). https://doi.org/10.1002/Aenm.201401377

[8] C. Chen, C. Zhao, Z. Hu, K. Liu, Synthesis of se/chitosan-derived hierarchical porous carbon composite as Li–Se battery cathode. Funct. Mater.Lett. 1650074 (2016). https://doi.org/10.1142/s1793604716500740

[9] C.P. Yang, S. Xin, Y.X. Yin, H. Ye, J. Zhang, Y.G. Guo, An advanced selenium-carbon cathode for rechargeable lithium-selenium batteries. Angew. Chem. Int. Edit. **52**(32), 8363-8367 (2013). https://doi.org/10.1002/anie.201303147

[10] S. Jiang, Z. Zhang, Y. Lai, Y. Qu, X. Wang, J. Li, Selenium encapsulated into 3D interconnected hierarchical porous carbon aerogels for lithium–selenium batteries with

high rate performance and cycling stability. J. Power Sources **267**, 394-404 (2014). https://doi.org/10.1016/j.jpowsour.2014.05.116

[11] Z. Li, L. Yuan, Z. Yi, Y. Liu, Y. Huang, Confined selenium within porous carbon nanospheres as cathode for advanced li–se batteries. Nano Energy **9**, 229-236 (2014). https://doi.org/10.1016/j.nanoen.2014.07.012

[12] J. Guo, Q. Wang, C. Qi, J. Jin, Y. Zhu, Z. Wen, One-step microwave synthesized core-shell structured selenium@carbon spheres as cathode materials for rechargeable lithium batteries. Chem. Commun. **52**(32), 5613-5616 (2016). https://doi.org/10.1039/c6cc00638h

[13] Y. Jiang, X. Ma, J. Feng, S. Xiong, Selenium in nitrogen-doped microporous carbon spheres for high-performance lithium–selenium batteries. J. Mater. Chem. A **3**(8), 4539-4546 (2015). https://doi.org/10.1039/c4ta06624c

[14] Y. Qu, Z. Zhang, S. Jiang, X. Wang, Y. Lai, Y. Liu, J. Li, Confining selenium in nitrogen-containing hierarchical porous carbon for high-rate rechargeable lithium–selenium batteries. J. Mater. Chem. A **2**(31), 12255 (2014). https://doi.org/10.1039/c4ta02563f

[15] Y. Liu, L. Si, Y. Du, X. Zhou, Z. Dai, J. Bao, Strongly bonded selenium/microporous carbon nanofibers composite as a high-performance cathode for lithium–selenium batteries. J. Phys. Chem. C **119**(49), 27316-27321 (2015). https://doi.org/10.1021/acs.jpcc.5b09553

[16] J.T. Lee, H.M. Kim, D-C. Lee, F. Wu et al., Micro- and mesoporous carbide-derived carbon-selenium cathodes for high-performance lithium selenium batteries. Adv. Energy Mater. **5**(1), 1400981-1400987 (2015). https://doi.org/10.1002/aenm.201400981

[17] K. Han, Z. Liu, J. Shen, Y. Lin, F. Dai, H. Ye, A free-standing and ultralong-life lithium-selenium battery cathode enabled by 3D mesoporous carbon/graphene hierarchical architecture. Adv. Funct. Mater. **25**(3), 455-463 (2015). https://doi.org/10.1002/adfm.201402815

[18] C. Luo, J. Wang, L. Suo, J. Mao, X.Fan, C. Wang, In situ formed carbon bonded and encapsulated selenium composites for Li-Se and Na-Se batteries. J. Mater. Chem. A **3**(2), 555-561 (2015). https://doi.org/10.1039/c4ta04611k

[19] J. Li, X. Zhao, Z. Zhang, Y. Lai, Facile synthesis of hollow carbonized polyaniline spheres to encapsulate selenium for advanced rechargeable lithium–selenium batteries. J. Alloys Compd. **619**, 794-799 (2015). https://doi.org/10.1016/j.jallcom.2014.09.099

[20] H.Ye, Y-X. Yin, S-F. Zhang, Y-G. Guo, Advanced Se–C nanocomposites: A bifunctional electrode material for both Li-Se and Li-ion batteries. J. Mater. Chem. A **2**(33), 13293 (2014). https://doi.org/10.1039/c4ta02017k

[21] Y. Lai, Y. Gan, Z. Zhang, W. Chen, J. Li, Metal-organic frameworks-derived mesoporous carbon for high performance lithium–selenium battery. Electrochim. Acta. **146**, 134-141 (2014). https://doi.org/10.1016/j.electacta.2014.09.045

[22] Z. Zhang, Z. Zhang, K. Zhang, X.Yang, Q. Li, Improvement of electrochemical performance of rechargeable lithium–selenium batteries by inserting a free-standing carbon interlayer. RSC Adv. **4**(30), 15489 (2014). https://doi.org/10.1039/c4ra00446a

[23] X. Peng, L. Wang, X. Zhang, B. Gao, J. Fu et al., Reduced graphene oxide encapsulated selenium nanoparticles for high-power lithium–selenium battery cathode. J. Power Sources **288**, 214-220 (2015). https://doi.org/10.1016/j.jpowsour.2015.04.124

[24] R. Fang, G. Zhou, S. Pei, F. Li, H-M. Cheng, Localized polyselenides in a graphene-coated polymer separator for high rate and ultralong life lithium–selenium batteries. Chem. Commun. **51**(17), 3667-3670 (2015). https://doi.org/10.1039/c5cc00089k
